# Social and Environmental Determinants of Diarrheal Diseases among Children under Five Years in Epworth Township, Harare

**DOI:** 10.3390/children10071173

**Published:** 2023-07-06

**Authors:** Sandra Chari, Thokozani Patrick Mbonane, Renay Helouise Van Wyk

**Affiliations:** Department of Environmental Health, Faculty of Health Sciences, University of Johannesburg, Johannesburg 2000, South Africa; 221183377@student.uj.ac.za (S.C.); renayvw@uj.ac.za (R.H.V.W.)

**Keywords:** social, environmental, determinants, diarrheal diseases, children, low- and middle-income countries

## Abstract

Children five years or younger in low- and middle-income countries (LMICs) are severely affected by diarrheal disease, especially in the sub-Saharan region. Hence, this study aimed at determining the prevalence and determinants of diarrhoea disease among children under 5 years in Epworth Township, Zimbabwe. A descriptive cross-sectional study was conducted at a local clinic in Epworth Township, Harare. A convenience sampling strategy was used to recruit study participants for participation, and 386 children were enrolled in the study. The majority were male children (*n* = 229; 59.3%), whereas there were more female caregivers (*n* = 370; 95.9%) than male caregivers (*n* = 16; 4.1%). The prevalence of diarrhoea disease in the study was 25.1%. The determinants associated with diarrhoea were being partially vaccinated (AOR 2.38, CI: 95% 2.80–8.22), collecting water more than 1 kilometre from a household (AOR 4.55; CI: 95% 2.10–9.85), and using untreated water (AOR 6.22; CI: 95% 2.13–18.20). The age of the caregiver (being older than 21) and using a clean water container (AOR 0.05; CI: 95% 0.02–0.13) were protective factors. Provision of primary health care, especially the prevention of a disease through immunization and rendering environmental health services, could reduce the prevalence of diarrhoea in disadvantaged townships.

## 1. Introduction

Diarrhoea is one major preventable source of childhood disease and mortality worldwide, especially in low-and middle-income countries (LMICs) [1]. According to the World Health Organization, it is estimated that there are 1.7 billion cases of diarrheal disease reported annually among children under 5 years [2]. The sub-Saharan region is the most affected in the world. Diarrheal disease accounts for 10–15% of deaths among children under 5 years old annually in the world and in Zimbabwe [3].

In low- and middle-income countries, diarrhoea is associated with environmental determinants, socio-economic status, and lack of access to primary health services [4]. Scientific evidence shows that a lack of sanitation facilities, urbanization, clean drinking water, proper waste disposal (including sewage), and living conditions contribute to the occurrence of diarrheal diseases. Furthermore, social determinants such as household economic status, parents’ characteristics, and behaviour have been linked to diarrheal disease [5,6].

Diarrheal diseases can cause a significant financial burden on households and the health care system. It is estimated that caregivers can incur costs ranging from USD 26 to USD 136 for a child diagnosed with diarrheal disease [7]. Children from low- and middle-income countries are the most affected by this condition. Childhood diarrheal disease may lead to severe health effects, such as stunted physical growth, cognitive impairment, malnutrition, and death, especially in LMICs such as Zimbabwe [8,9,10].

Primary prevention strategies such as water, sanitation, and hygiene (WASH) interventions can reduce the risks and incidences of diarrhoea among children [11]. This includes the provision of adequate safer water infrastructure and the administering of the rotavirus vaccine [12]. Such interventions are recommended by the Sustainable Development Goal (SDG) 6. Children infected with diarrhoea can be treated in numerous various methods (such as oral rehydration solutions, antibiotic treatments, immunization, and feeding practices) to prevent high mortalities and morbidities [7]. In Zimbabwe, the lack of basic sanitation, water infrastructure, and the non-availability of the rotavirus vaccine has resulted in a significantly increased incidences of diarrhoea cases [13]. A study conducted in Zimbabwe in 2019 showed a decrease in hospitalization due to severe rotavirus among young children [14].

There is a high rotavirus vaccine dropout and a low adherence in most LMICs [15]. Furthermore, most parents do not take their children for medical attention, and most diarrhoea cases end up not being diagnosed in the Sub-African region, including Zimbabwe [2]. The objectives were to describe the prevalence of diarrheal diseases in children under five in Epworth and establish the social and environmental determinants of diarrhoea diseases in children under five in Epworth.

## 2. Materials and Methods

### 2.1. Study Setting and Population

A descriptive cross-sectional study was conducted in a local clinic of the Epworth Township (characterized by an informal settlement setting), Harare, from July to September 2022. In this study, children aged 0 to 5 years presenting with complaints of diarrhoea were targeted. Epworth Township is a highly dense impoverished township with limited primary health services, access to water, sewage services, and cleaner energy [16]. The population is estimated to be 167,462, according to the 2012 census survey [16]. The township and study population were selected because of the living conditions, lack of access to services, and diarrheal cases reported in other studies with similar conditions. The targeted study population comprised children under the age of 5 years that were presented to the local clinic.

### 2.2. Study Inclusion and Exclusion Criteria

Study participants were included in the study if they met the following: (i) a mother or caregiver was present; and (ii) had one or more children under the age of five. In case the mother or caregiver presented with one or more children, all were invited to participate, whereas mothers or caregivers who were mentally ill, seriously ill, and under 18 years were excluded from the study. Furthermore, returning patients (children) that had been previously enrolled were excluded in the study.

### 2.3. Sampling and Sample Size

A convenient sampling strategy was implemented to recruit participants for study participation. Caregivers presenting with children under the age of 5 years at a local clinic were approached until the sample size was met, and only a mother/caregiver or guardian older than 18 years was interviewed. The sample size was estimated using Epi Info 7.20, with the assumption of a 21.5% prevalence of diarrhoea amongst children under 5 years in the target study site with a 5% margin of error, 95% confidence level, and 5% standard deviation based on a study conducted elsewhere. The ratio between those with diarrhoea and no diarrhoea was set at 1:1. Therefore, the estimated sample size was 395.

### 2.4. Socio and Environmental Determinants

A structured questionnaire administered by trained research assistants was used to collect data on social and environmental determinants in this study. The questionnaire was designed in English and translated into Shona, the local language, and vice versa. It was used to collect the following information: participants’ socio-demographic characteristics (including caregivers’ details), the prevalence of diarrhoea, participants’ behaviours, environmental factors, and social determinants status. The questionnaire was piloted in a similar setting to assess its validity, duration of completion, and participants’ understanding of the questions. A test re-test reliability was used to ensure the reliability of the questionnaire.

### 2.5. Outcome Variable

The study outcome variable was diarrhoeal disease among children under five years. In this study, diarrhoea was defined as having experienced the following symptoms in the last 14 days: passing three or more liquid/loose stools, and bowel movements in a 24 h period.

### 2.6. Vaccination and Nutritional Status

#### 2.6.1. Vaccination Status

The Rotavirus vaccine is administered in two doses at different ages. For the purpose of this study, those that received two doses of the rotavirus were classified as fully vaccinated. Those that received one dose were partially vaccinated. Lastly were the children that never received any vaccine for diarrhoea.

#### 2.6.2. Nutritional Status

The participants’ Mid-Upper Arm Circumferences (MUACs), weights, and heights were measured. The MUAC was used as a nutritional indicator, forming part of the health screening. It was then categorized as (1) less than 12 and (2) 12 and above.

### 2.7. Data Analysis

The collected data were captured, cleaned, coded, and analysed using IBM SPSS version 27. Descriptive statistics (frequencies and percentages) were used to describe the distributions of demographic characteristics, behavioural patterns, social characteristics, and socio-economic and environmental factors. The binary logistic regression model was adopted. Variables (determinants) that were statistically significant in the bivariate analyses were included in the final model. A backward likewise multivariate binary logistics regression was used to determine the effect of determinants on diarrheal cases. The significance was set at *p* < 0.05.

### 2.8. Ethical Considerations

The study obtained ethical clearance from the University of Johannesburg Research Ethics Committee (REC-1654-2022). Informed consent was obtained from the mother/caregiver before commencing the study.

## 3. Results

### 3.1. Study Participants’ Socio-Demographic Characteristics

There were 386 participants that participated in the study. Most of the children were aged between 12–22 months (32.4%, *n* = 125) in this study. There were more male children (59.3%, *n* = 229) than female children (40.7%, *n* = 157). Most of the children had no chronic illness (99.5%, *n* = 384), and 2 (0.5%) children were recorded to have known chronic illness. Mid-upper arm circumference (MUAC) was used for the assessment of the nutritional status of the children, and 68 (17.6%) children had a MUAC of less than 12. Most of the caregivers were female (95.9%, *n* = 370). The majority of them were aged between 18–30 years (78.5%, *n* = 303). In this study, 83.9% (*n* = 324) of the caregivers indicated that they were the child’s mother, and 1.6% (*n* = 6) indicated that they were the child’s father. Looking at the highest level of education of the caregivers, 140 (36.3%) had secondary school education. In this study, most households earned between 50 and 100 USD per month (52.6%, *n* = 203). Lastly, most caregivers indicated that there were 2 or more children under 5 years (59.3%, *n* = 229) in their household. Most of the children were fully vaccinated with the rotavirus vaccine (61.1%, *n* = 236), whereas 132 (34.2%) were partially vaccinated (received one dose of the rotavirus). Table 1 shows detailed characteristics of the children and caregivers, as well as behavioural factors.

### 3.2. Prevalence of Diarrheal l Diseases

There were 97 (25.1%) children who had experienced diarrhoea, and 74.9% did not experience diarrhoea. Therefore, the prevalence of diarrhoea in this study was 25.1%. Participants (*n* = 97; 25.1%) that reported having experienced diarrhoea were asked about condition duration, stool characteristics, treatment, and type of treatment received, as shown in Appendix A (Table A1). Further analysis showed that 72.2% (*n* = 70) reported to have had watery diarrhoea, and 20.6% (*n* = 27) had mucoid diarrhoea. Regarding the duration of diarrhoea, most of the children had diarrhoea for less than 3 days (*n* = 70; 72.2%). The survey indicated that most participants (*n* = 93; 95.9%) sought treatment from a health facility. The most treatment received by children who had experienced diarrhoea was oral rehydration therapy (*n* = 91; 93.8%).

### 3.3. Social and Environmental Related Determinants

Households with more than four people had a high number of diarrheal cases (*n* = 205; 70.9%). There were diarrheal cases from households that used a community borehole (*n* = 51; 52,6%), collected water in a distance of more than 1 km (*n* = 62; 63.9%), and spent more time collecting water (*n* = 75; 77.3%). The bivariate analysis showed statistical significance when comparing participants with diarrhoea and those with no diarrhoea for the following determinants (*p* < 0.001): drinking water collected outside the household (*p* < 0.001), untreated water (*p* < 0.001), sharing a toilet (*p* = 0.027), and using a toilet with no hygiene facilities (*p* < 0.001), as shown in Table 2.

### 3.4. Determinants Influencing Diarrhoea in the Study Population

The multivariate analysis (as presented in Table 3) showed that diarrhoea was associated with being unvaccinated (*p* = 0.022), the households that collected water at a distance, and using untreated water (*p* < 0.001). However, ages of the caregivers (21–30 years old (COR: 0.22; 95% CI 0.12–0.40), 31–40 years old (*p* < 0.001) and 41–50 years old (*p* = 0.007), (*p* < 0.001), using clean water containers (COR: 05; 95%CI 0.02–0.13), were protective factors in the study.

## 4. Discussion

This study aimed to determine the prevalence of diarrhoea and associated factors among Epworth’s under-five children. In this study, the prevalence of childhood diarrheal diseases was 25.1% over two weeks. This finding was higher than studies conducted elsewhere in the Southern African Development Community (SADC) region [6,18]. A study conducted in a South African low-and middle-income township (Soweto) found a diarrheal disease prevalence of 20.9% among children under 5 years old [18]. This study’s prevalence was higher when compared to a study in Mozambique with a similar population that found a prevalence of 10.6% in the 10,026 children under the age of five [6]. However, it was lower when compared to a conducted in northern Nigeria, with a prevalence of 37.7% [19]. In the areas where the prevalence was low, the communities had access to basic needs, such as access to water, which was not the same in the Epworth Township. This could explain the variation between the different sites.

In this study, having an older caregiver and using a clean water container were protective factors. This study found that being born to an older mother was a protective factor. The findings were consistent with previous studies. A study in Nigeria found that children born to mothers aged 25–34 were 15% less likely to have diarrhoea than children born to mothers aged 15–24 [20]. It is believed that older women may have experience in childcare and knowledge about diarrheal disease, its mode of transmission, and risk factors associated with diarrhoea [21]. Thus, as it is an important find for preventing diarrhoea in children under the age of five, health education interventions should include young mothers as one of the target audiences.

Children that were partially vaccinated were at a higher risk (AOR 2.38; 95% CI: 1.08–5.25) of suffering from diarrheal disease. This finding was important as it highlighted the impacts of being vaccinated in preventing diarrheal disease in low- and middle-income countries [22]. A long-term study in Fiji showed a reduction of 81% in diarrhoea mortality cases among children under 5 years [23]. The decline was due to the rotavirus vaccine. Therefore, healthcare workers and policymakers need to ensure access to preventive care for the protection of vulnerable groups such as children under 5 years old.

Children from households who travelled more than a kilometre to water sources were 3.55 times more probable to have diarrhoea than those children from households who travelled more than a kilometre. Our findings were similar to a study that reported that distance to water sources showed a strong association with under-five childhood diarrhoea morbidity [24]. This could be because most water sources near households are shallow wells that risk being contaminated by faecal material. Lastly, using untreated water was a risk factor (*p* > 0.001) in the study. This has been proven in previous studies [25,26,27,28,29], as contaminated water collected in shallow wells (*n* = 136; 35.2%), community boreholes (*n* = 227; 58.8%), and personal boreholes (*n* = 13; 3.4%) was neither chlorinated nor boiled before use. There is a need for environmental health services to ensure preventive measures such as the provision of safe water and health education on how to clean water before use. A study conducted in India also highlighted the importance of addressing adverse living environmental factors [30].

## 5. Strength and Limitations

The key strength of the study was that the respondents were obtained from the local clinic, making it simple and efficient. Hence, the study was quick to conduct. Therefore, the research question was addressed in a short space of time. The data in this study were gathered using a cross-sectional survey, which only represented a part of the population of Epworth at that particular time with only the individuals that used that particular clinic, and there were no follow-ups. It was difficult to account for seasonal variations in the occurrences of child diarrhoea because the predictor variables and the outcome variable were measured at the same time. Lastly, the study could not be generalized to other townships in Zimbabwe.

## 6. Conclusions

Environmental and infrastructural deficits, such as water accessibility, the presence and use of latrines, the availability of hand washing facilities, and waste disposal methods, are the major determinants of diarrhoea. As a result, there is a need to improve access to these facilities and health education awareness on the prevention of diarrhoea in Epworth through an integrated and comprehensive approach to reducing diarrheal-related morbidity and mortality among children under the age of five.

## Figures and Tables

**Table 1:** Detailed Description of Socio-demographic Characteristics.

Characteristics	Frequency (*n*)	Percentage (%)
Child Characteristics		
Child Gender		
Male	229	59.3%
Female	157	40.7%
Child Age		
0–11 months	103	26.7%
12–23 months	125	32.4%
24–35 months	49	12.7%
36–47 months	85	22%
48–59 months	24	6.2%
Chronic illness		
No	384	99.5%
Yes	2	0.5%
MUAC		
Less than 12	68	17.6%
Above 12	318	82.4%
Caregiver Characteristics		
Gender of caregiver		
Male	16	4.1%
Female	370	95.9%
Age of caregiver		
18–20	165	42.7%
21–30	138	35.8%
31–40	56	14.5%
41–50	27	7%
Relationship with child		
Mother	324	83.9%
Father	6	1.6%
Grandparent	29	7.5%
Aunt/Uncle	2	0.5%
Other	25	6.5%
Highest level of education		
Uneducated	92	23.8%
Primary School	115	29.8%
Secondary school	140	36.3%
Diploma	33	8.5%
Degree	6	1.6%
Family Income *		
Less than 50	170	44%
50 to 100	203	52.6%
100 to 200	11	2.8%
Above 200	2	0.5%
Number of children under 5 years		
One	157	40.7%
Two	213	55.2%
3	16	4.1%
Behaviour		
Child weaning age		
Less than 6 months	47	12.2%
6–18 months	185	47.9%
19–24 months	34	8.8%
25–36 months	9	2.3%
Still Breastfeeding	111	28.8%
Vaccination Status		
Full Vaccinated	236	61.1%
Unvaccinated	18	4.7%
Partially vaccinated	132	34.2%

* US dollar currency was used, as it is the current preferred currency of trade in Zimbabwe [17].

**Table 2:** Social and Environmental related determinants in the Study.

Determinants		Diarrhoea		Total *n* (%)	Chi-Squared *p*-Value
		Yes *n* (%)	No *n* (%)		
Number of people per household?	2	6 (2.1%)	-	6 (1.6%)	0.334
	3	28 (9.7%)	8 (8.2%)	36 (9.3%)	
	4	50 (17.3%)	13 (13.4%)	63 (16.3%)	
	More than 4	205 (70.9%)	76 (78.4%)	281 (72.8%)	
What is the main water source?	Shallow well	43 (44.3%)	93 (32.3%)	136 (35.2%)	0.105
	Community borehole	51 (52.6%)	176 (61%)	227 (58.8%)	
	Personal borehole	1 (1%)	12 (4.2%)	13 (3.4%)	
	Council tapped water	2 (2.1%)	8 (2.8%)	10 (2.6%)	
Covered water container?	Yes	97 (100.0%)	289 (100.0%)	386 (100%)	
Distance to water source?	Within household	35 (36.1%)	187 (64.8%)	222 (57.5%)	<0.001 *
	More than 1 km	62 (63.9%)	102 (35.3%)	164 (42.5%)	
Time spent collecting water?	30 min	22 (22.7%)	67 (23.2%)	89 (23.1%)	0.919
	1 h	75 (77.3%)	222 (76.9%)	297 (76.9%)	
How is water drawn from storage container	Dipping scooper	32 (33%)	87 (30.1%)	119 (30.8%)	0.594
	Pouring out	65 (67%)	202 (70%)	267 (69.2%)	
Do you normally empty/clean containers?	Yes	58 (59.8%)	133 (46.1%)	191 (49.5%)	0.019
	No	39 (40.2%)	156 (54%)	195 (50.5%)	
Is the water treated for drinking?	Yes	45 (46.4%)	233 (80.6%)	278 (72%)	<0.001 *
	No	52 (53.6%)	56 (19.5%)	108 (28%)	
How is water treated?	Chlorination	15 (15.5%)	62 (21.6%)	77 (19.9%)	<0.001 *
	Boiling	31 (32%)	171 (59.2%)	202 (52.3%)	
	No treatment	51 (52.6%)	56 (19.5%)	107 (27.7%)	
Is there a toilet?	Yes	97 (100%)	289 (100%)	386 (100%)	
Is the toilet shared?	Yes	81 (83%)	209 (72.3%)	290 (72.3%)	0.027 *
	No	16 (16%)	80 (27.7%)	96 (27.7%)	
How many times is the latrine cleaned in a week?	Daily	97 (100%)	286 (99.0%)	383 (99%)	0.602
	2–3 times	-	1 (0.3%)	1 (0.3%)	
	4–6 times	-	2 (0.7%)	2 (0.7%)	
Are there hand washing facilities with soap near the toilet?	Yes	15 (15%)	186 (64.4%)	201 (64.4%)	<0.001 *
	No	82 (84%)	103 (35.6%)	185 (35.6%)	

* *p*-value significant at 0.050.

**Table 3:** Logistic regression model determinants of diarrheal disease among under-five children.

Determinants		Bivariate Model		Multivariate Model	
		COR (95% CI)	*p*-Value	AOR (95% CI)	*p*-Value
Vaccination status of child	Fully Vaccinated	Ref			
	Unvaccinated	0.80 (0.32–2.03)	0.003	1.32 (0.39–4.41)	0.402
	Partially vaccinated	0.67 (0.47–0.95)	<0.001 *	2.38 (1.08–5.25)	0.022 *
Age of caregiver	16–20	Ref			
	21–30	0.22 (0.14–0.34)	<0.001 *	0.22 (0.12–0.40)	<0.001 *
	31–40	0.06 (0.02–0.18)	<0.001 *	0.06 (0.02–0.23)	<0.001 *
	41–50	0.13 (0.04–0.42)	0.008 *	0.10 (0.02–0.44)	0.007 *
Distance to water	Within household	Ref			
	More than 1 km	0.61 (0.44–0.83)	<0.001 *	4.55 (2.10–9.85)	<0.001 *
Clean container usage	No	Ref			
	Yes	0.25 (0.18–0.36)	0.020 *	0.05 (0.02–0.13)	<0.001 *
Treatment method	Chlorination	Ref			
	Boiling	0.18 (0.12–0.27)	0.407	0.34 (0.18–0.63)	0.194
	No treatment	0.91 (0.62–1.33)	<0.001	6.22 (2.13–18.20)	0.001 *

* *p*-value significant at 0.050.

## Data Availability

The data presented in this study are available on request from the corresponding author. The data are not publicly available due to ethical reasons.

## Appendix A

**Table A1:** Further analysis of diarrheal cases reported in the study.

Diarrhoea Signs and Actions		Frequency (*n*)	Percentage (%)
How long has child had diarrhoea?	Less than 3 days	70	72.2%
	4–7 days	20	20.6%
	8–14 days	7	7.2%
The diarrhoea is generally:	Watery	70	72.2%
	Mucus and Bloody	27	27.8%
Was treatment sought?	Yes	96	99%
	No	1	1.%
Where was treatment sought?	Health Facility/Clinic/Hospital	93	95.9%
	Pharmacy	2	2.1%
	At Home	2	2.1%
What treatment did they receive?	Oral Rehydration Therapy	91	93.8%
	Other Medication	5	5.2%
	Home Remedies	1	1%
